# Breaking the symmetry to suppress the Plateau–Rayleigh instability and optimize hydropower utilization

**DOI:** 10.1038/s41467-021-27237-0

**Published:** 2021-11-25

**Authors:** Zhipeng Zhao, Huizeng Li, An Li, Wei Fang, Zheren Cai, Mingzhu Li, Xiqiao Feng, Yanlin Song

**Affiliations:** 1 Key Laboratory of Green Printing, Institute of Chemistry, Chinese Academy of Sciences (ICCAS)/Beijing Engineering Research Center of Nanomaterials for Green Printing Technology, Beijing National Laboratory for Molecular Sciences (BNLMS), Beijing, 100190 People’s Republic of China; 2 University of Chinese Academy of Sciences, Beijing, 100049 People’s Republic of China; 3 AML, CNMM and Department of Engineering Mechanics and State Key Laboratory of Tribology, Tsinghua University, Beijing, 100084 People’s Republic of China

**Keywords:** Fluid dynamics

## Abstract

Droplet impact on solid surfaces is essential for natural and industrial processes. Particularly, controlling the instability after droplet impact, and avoiding the satellite drops generation, have aroused great interest for its significance in inkjet printing, pesticide spraying, and hydroelectric power collection. Herein, we found that breaking the symmetry of the droplet impact dynamics using patterned-wettability surfaces can suppress the Plateau–Rayleigh instability during the droplet rebounding and improve the energy collection efficiency. Systematic experimental investigation, together with mechanical modeling and numerical simulation, revealed that the asymmetric wettability patterns can regulate the internal liquid flow and reduce the vertical velocity gradient inside the droplet, thus suppressing the instability during droplet rebounding and eliminating the satellite drops. Accordingly, the droplet energy utilization was promoted, as demonstrated by the improved hydroelectric power generation efficiency by 36.5%. These findings deepen the understanding of the wettability-induced asymmetrical droplet dynamics during the liquid–solid interactions, and facilitate related applications such as hydroelectric power generation and materials transportation.

## Abstract (web summary)

Satellite drops formed on droplet impact hinder applications from inkjet printing to drop energy harvest. Here, authors reveal that patterned-wettability surfaces can break the symmetry of droplet impacts and suppress the Plateau–Rayleigh instability, preventing satellite drop generation and improving the hydropower harvest.

## Introduction

Droplet impact on solid surfaces has been extensively investigated owing to its significance in various fields such as hydroelectric power collection^1–4^, inkjet printing^5–8^, and anti-icing^9–14^. A very essential branch is the investigation of the droplet instability suppression after impacting on various surfaces. Two types of instability exist during the droplet impact. One is the splashing that is dominated by the Kelvin–Helmholtz instability in the droplet spreading stage, where the thin air layer between the droplet and the solid is compressed and induces splashing at the edge of spreading droplet under certain criteria, thus producing satellite drops^15–19^. The other is the Plateau–Rayleigh instability in the droplet retraction stage^13,20–23^. The retracting liquid at the periphery of the droplet collides and generates an upward liquid column, which will excessively elongate and finally break up into satellite drops under slight disturbances.^17,23^ The Plateau–Rayleigh instability is one of the most widely recognized phenomena during droplet retraction process, and has adverse effects on inkjet printing^24^, pesticide spraying^15,25^, droplet-based energy collection^26^, and many other applications^20,27–33^.

Great efforts have been made to suppress the instability after droplet impacting on the solid surface and the associated satellite drops formation. For example, vesicle surfactants were added into water droplets to prevent the droplet splashing when pesticide spraying^15^; and micro-holes array was fabricated on the substrate to restrain the Kelvin–Helmholtz instability during droplet impacting^16^. However, it is still challenging to regulate the Plateau–Rayleigh instability during droplets rebounding on solid surfaces due to the fast retraction velocity of the droplet and the excessive velocity gradient inside the droplet, which elongate the liquid column and induce the generation of satellite drops.

Regulating surface wettability has proven to be an effective method for precisely controlling the droplet dynamic behaviors^34,35^. For example, the gyrating and dancing of droplets are realized through heterogeneous surface wettability regulation^33^, and droplet directional bouncing and materials transportation^36–39^ have been achieved by constructing patterned-wettability surface where the droplet lateral velocity is found to be related to the surface area of a geometric region^36,37^. During these rebounding processes, however, satellite drops may still be produced due to Plateau–Rayleigh instability. Up to now, the mechanism and principle for precisely regulating the droplet Plateau–Rayleigh instability behavior remain unclear. Herein, we propose that using patterned-wettability substrates, the symmetry of the droplet impact dynamics can be broken, which suppresses the Plateau–Rayleigh instability during the droplet rebounding process, and improves the hydroelectric energy collection efficiency. It is demonstrated that the Plateau–Rayleigh instability can be evaluated by the droplet elongation and the formation of satellite drops. Meanwhile, it is negatively correlated with the main droplet rebounding kinetic energy. From both experimental and theoretical aspects, we uncover the mechanism of suppressing instability during droplet rebounding, that the asymmetric superhydrophilic pattern on the superhydrophobic surface regulates the liquid flow and reduces the vertical velocity gradient inside the droplet. As a result, the instability is suppressed, with the shortened droplet elongation and the suppressed satellite drops. On this basis, the hydroelectric energy collection efficiency of a wettability-patterned device can be promoted by 36.5% compared with that of a superhydrophobic device. These findings provide insights into the asymmetrical liquid–solid dynamics, and inspire the innovative design of functional surfaces for liquid instability suppression, high-performance hydroelectric energy collection, and droplet directional transportation.

## Results

### Suppress the Plateau–Rayleigh instability by breaking the symmetry of surface wettability

**Fig. 1:**
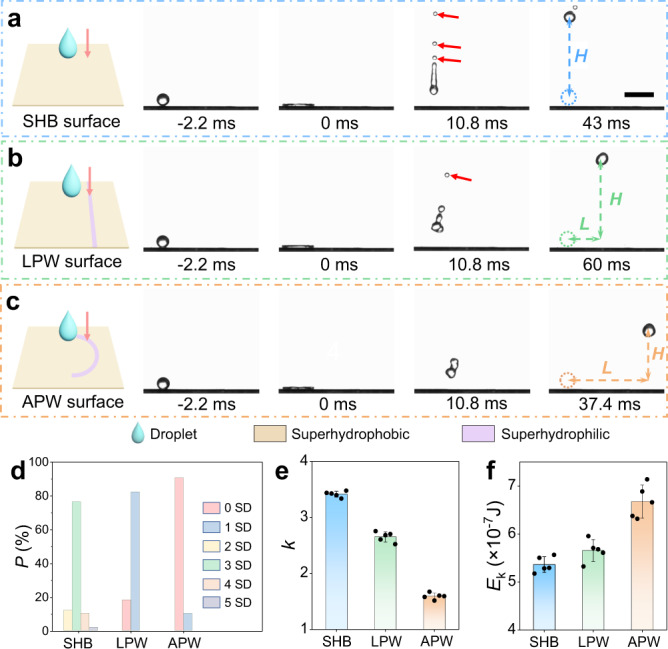
Retraction of the droplets impacting on substrates with diverse wettability. **a**–**c** Side view of the water droplets impacting on the superhydrophobic (SHB) surface, the line-patterned-wettability (LPW) surface, and the arc-patterned-wettability (APW) surface. Scale bar: 5 mm. *W*e = 32.8. **d** Probability distribution of the satellite drop (SD) number of 3 surfaces (50 impacts for each surface). **e** *k* (defined as the ratio of droplet elongation length and droplet diameter *D*) of the 3 surfaces. **f** The rebounding kinetic energy *E*_k_ of the main droplets of the 3 surfaces. The error bars of the data in **e**, **f** are obtained from the s.d. (standard deviation) of 5 independent experiments.

We prepare superhydrophobic (SHB) substrates, and design superhydrophilic patterns on the substrates to form wettability patterns. The SEM characterization of the substrate is shown in Supplementary Fig. 1. The surface with a line-shaped superhydrophilic pattern is termed as line-patterned-wettability (LPW) surface, and the surface with an arc-shaped superhydrophilic pattern is termed as arc-patterned-wettability (APW) surface. Note that for the LPW surface, the line is deviated from the substrate center, while for the APW surface, the centers of the patterns and the substrates are overlapped. Then water droplets are released to impact on the center of the substrates at *We* = 32.8, where *We* = $\rho {v}^{2}R/\gamma$ is the Weber number, with $\rho$ being the density, $v$ being the impacting velocity, *R* being droplet radius, and $\gamma$ the surface tension of water (see Supplementary Movie 1). Selected snapshots of the droplet evolution on these surfaces are shown in Fig. 1a–c. The droplet rebounds vertically upward and generates 3 satellite drops after impacting on the SHB surface (Fig. 1a). In contrast, the droplets rebound upward with lateral deviation on the LPW surface with 1 satellite drop and APW surface with 0 satellite drop (Fig. 1b, c). To further confirm the different numbers of the satellite drops, we perform 50 independent droplet impacting tests on each of the three surfaces. The results in Fig. 1d show that there is an 88% probability that the droplet will produce at least 3 satellite drops when impacting on the SHB surface, 82% probability of droplet producing 1 satellite drop on the LPW surface and 90% probability of droplet producing 0 satellite drop on the APW surface. It indicates that the different numbers of the satellite drop in Fig. 1a–c are not accidental. Meanwhile, we find that the stretching length of the main droplet at the moment of separating from the surface varies greatly on different surfaces. The droplet elongation is the largest on the SHB surface, followed by the LPW and the APW surfaces, as shown in Fig. 1e.

The droplet elongation and the satellite drops generation are originated from the instability during droplet rebounding. On the one hand, due to the droplet elongation, more surface energy is converted from kinetic energy, while the increased surface energy is fully dissipated during the droplet rebounding (detailed discussion is provided in Supplementary Method 1). As a result, the droplet elongation shows a negative effect on the main droplet kinetic energy. On the other hand, the generation of satellite drops reduces the main droplet kinetic energy (see Supplementary Fig. 2). Therefore, we can use the kinetic energy *E*_k_ of the main droplet to evaluate the instability during droplet rebounding. Figure 1f illustrates the *E*_k_ on the SHB, LPW, and APW surfaces. Detailed calculation process is provided in Supplementary Method 1. Compared with the kinetic energy of the main droplet on the SHB surface, the LPW and APW surfaces show 5.4% and 24.6% improvement, respectively, which is consistent with the analysis above. The results indicate that the Plateau–Rayleigh instability during droplet rebounding can be suppressed by the wettability patterns on the LPW and the APW surfaces. As the droplet rebounding kinetic energy is related to the liquid flow inside the droplets^17,20^, we hypothesize that the instability suppression during droplet rebounding on the LPW and APW surfaces originates from the differentiated liquid flow fields.

### Characterization of asymmetrical liquid flow inside the droplet when retracting

**Fig. 2:**
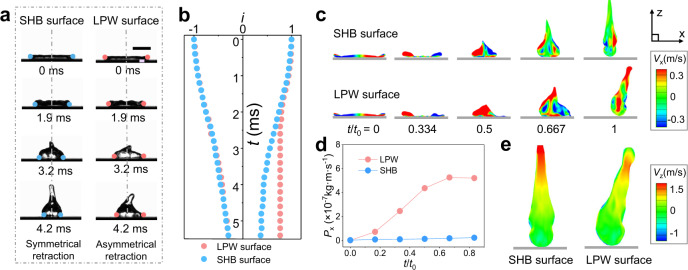
Dynamics of the asymmetrical and symmetrical droplets retraction. **a** Snapshots of the symmetrical droplet retraction after impacting on the SHB surface, and the asymmetrical droplet retraction after impacting on the LPW surface. *We* = 32.8. Scale bar: 2 mm. **b** Plot of the TCL position *i* (defined as the ratio of the position of the droplet’s ends and the droplet radii) of the symmetrical and asymmetrical retracting droplets. **c** Simulated results to show the evolution of the lateral (*x*-direction) velocity distribution in the cross-section of the droplets. **d** The momentum of the droplet along the *x*-direction (*P*_x_) during the retraction process. **e** The vertical velocity gradient (*z*-direction) inside the droplets at *t*/*t*_0_ = 1.

To investigate the liquid flow inside the droplets, we record the droplets evolution after impacting on the SHB and the LPW surfaces. Here the LPW surface is selected as the representative of patterned wettability surfaces. As the droplets show indistinguishable spreading behaviors on the surfaces (Supplementary Fig. 4), we mainly focus on the droplet retraction stage, as the sequenced images shown in Fig. 2a. The liquid film retracts symmetrically on the SHB surface, with synchronized and continuous three-phase contact line (TCL) dewetting on both sides. By contrast, the liquid film retracts asymmetrically on the LPW surface, with the left TCL freely dewetting while the right TCL pinned. The position evolution of the TCL on the two surfaces is shown in Fig. 2b. The curves overlap at the initial stage of retracting (0-1.9 ms), while noncoincidence appears when the right TCL on the LPW surface is captured by the superhydrophilic stripe. The dynamic contact angle and contact line velocity of the two surfaces are shown in Supplementary Fig. 5, Supplementary Discussion 1. According to the previous result^36,40,41^, the droplet on the LPW surface is subjected to an unbalanced lateral force *F*_Lat_^40,41^, which may affect the liquid flow inside the droplet. For clear clarification, we simulate the droplet impact process on these surfaces using the coupled Level-Set and Volume of Fluid (CLSVOF) method (Details see Supplementary Method 2). For the droplet symmetrically retracting on the SHB surface, the TCLs move simultaneously toward each other, forming opposite liquid flow on the two halves of the droplet (the top image in Fig. 2c, *t*/*t*_0_ = 0–0.5). Here *t*_0_ is the duration of the droplet retraction. While for the droplet asymmetrically retracting on the LPW surface, the obvious retarding of the right TCL results in a dominated asymmetrical liquid flow (the bottom image in Fig. 2c, *t*/*t*_0_ = 0–0.5). The droplet lateral momentum (*x*-direction) in the symmetrical and asymmetrical retraction stages are extracted and shown in Fig. 2d. The lateral momentum on the LPW surface gradually enlarges with droplet retracting, which is caused by the integration of unbalanced lateral force with time, while it remains zero on the SHB surface that shows little lateral force to the droplet. Meanwhile, as shown in Fig. 2e, when departing from the LPW surface, the vertical velocity gradient is smaller than that from the SHB surface, thus reducing the elongation of the droplet and suppressing the generation of satellite drops^17,20^.

**Fig. 3:**
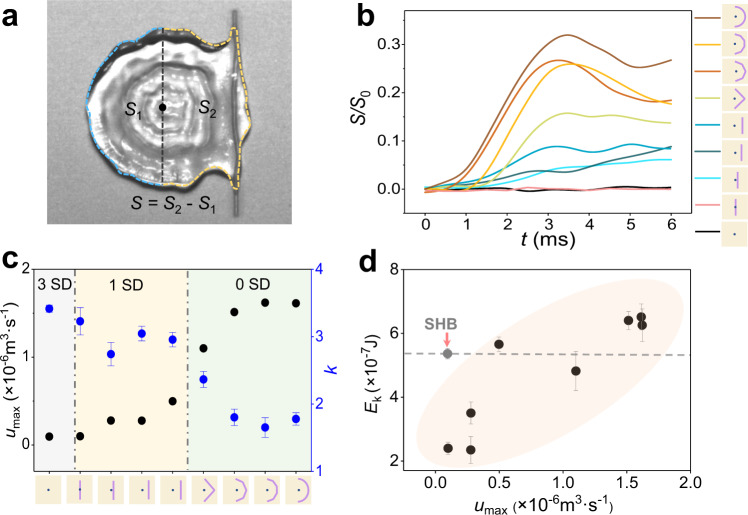
Effect of the liquid flow on the satellite drop number, elongation length and the main droplet kinetic energy. **a** The definition of area *S* at time *t*. **b** Changes of the area *S/S*_0_ with time *t* on different wettability surfaces. **c** Changes in the maximum net liquid flow rate *u*_max_, satellite drop number and *k* (defined as the ratio of droplet elongation length and droplet diameter *D*) on different wettability surfaces. **d** The influence of the maximum net liquid flow rate *u*_max_ on the kinetic energy of the main droplets *E*_k_. The error bars of the data in **c**, **d** are obtained from the s.d. of 5 independent experiments.

Next, the influences of the symmetry of the liquid flow on the rebounding droplet elongation and the satellite drop generation are quantitatively discussed. The character, *u*, is used to describe the net liquid flow rate through a specific cross-section, as indicated by the black dashed line in Fig. 3a. Here, we assume that the height *h* of the left and right parts of the droplet is the same. The net liquid flow rate can be expressed as $u={{{{{\rm{d}}}}}}V/{{{{{\rm{d}}}}}}t=S\times {{{{{\rm{d}}}}}}h/{{{{{\rm{d}}}}}}t+h\times {{{{{\rm{d}}}}}}S/{dt}$, where *V* is the net liquid flow, *h* is the thickness of the liquid film, and *S* is the bottom areas difference of the two parts (*S* = *S*_2_ – *S*_1_). According to Fig. 2a, *h* almost remains unchanged (0 ms < t < 3.2 ms). Hence, we get $u \sim {{{{{\rm{d}}}}}}S/{{{{{\rm{d}}}}}}t$, which means that the net liquid flow rate can be evaluated by the change rate of the bottom area difference *S*. Figure 3b plots the variation of the bottom area difference *S/S*_0_ on various surfaces (*S*_0_ is the bottom area of droplet maximum spreading), and the derivative of *S* with respect to time (${{{{{\rm{d}}}}}}S/{{{{{\rm{dt}}}}}}$) is shown in Supplementary Fig. 6. The maximum value ${({{{{{\rm{d}}}}}}S/{{{{{\rm{d}}}}}}t)}_{{{{{{\rm{max }}}}}}}$ represents the maximum bottom area difference change rate. The maximum net flow rate can be expressed as ${u}_{{{{{{\rm{max }}}}}}}=h\times {({{{{{\rm{d}}}}}}S/{{{{{\rm{d}}}}}}t)}_{{{{{{\rm{max }}}}}}}$, which represents the capability of the asymmetrical liquid flow of the droplets on these surfaces. Figure 3c shows the maximum net flow rate *u*_max_ of different wettability surfaces, as well as satellite drop number and *k* (defined as the ratio of droplet elongation length and droplet diameter *D*) on different wettability surfaces (The dynamic contact angle difference of the two ends of the droplet and the duration for the difference are shown in Supplementary Fig. 7). On the SHB surface (light gray area), the droplet possesses a small ${u}_{{{{{{\rm{max }}}}}}}$ and shows the largest elongation length, generating the most satellite drops. Increasing ${u}_{{{{{{\rm{max }}}}}}}$ using wettability-patterned substrates shortens the droplet elongation and suppresses the satellite drops generation (light yellow area). Using optimized wettability patterns, the satellite drops are fully suppressed (light green area). As ${u}_{{{{{{\rm{max }}}}}}}$ shows quantitative influence on the satellite drop number and the droplet elongation length, which are affected by the liquid flow fields inside the droplets, we investigate the dependence of the main droplet rebounding kinetic energy on ${u}_{{{{{{\rm{max }}}}}}}$. As shown in Fig. 3d, with the increase of ${u}_{{{{{{\rm{max }}}}}}}$, the droplet rebounding kinetic energy gradually increases, and becomes larger than that on the SHB surface. Note that the SHB surface has the smallest ${u}_{{{{{{\rm{max }}}}}}}$ but a relatively large kinetic energy, which can be explained that the adhesion force between the SHB surface and the droplet is negligible (Details see Supplementary Note 1). The results verify our hypothesis that Plateau–Rayleigh instability of rebounding droplets is dominated by the symmetry of the liquid flow field inside the droplet. In addition, the generality of suppressing Plateau–Rayleigh instability by breaking the symmetry of surface wettability is proved applicable to a wide range of *We* numbers and viscosities (Details see Supplementary Discussion 2).

### Application of breaking the symmetry of the droplet impact dynamics

Hydropower generation plays an increasingly important role in renewable energy. Compared with the well-established hydroelectric plants that need large amounts of water for electricity generation, the collection and utilization of energy from more accessible forms of water, such as raindrops, is also important but usually underestimated^1,4,42–44^. Recently, the innovation of triboelectric and piezoelectric generators has boosted the development of energy collection from raindrops^2,4,45–49^. For the piezoelectric generator, when an external force is applied, the change in the structure of the piezoelectric materials induces the formation of an electric dipole, which produces a voltage across the device. After a water droplet impacting on the piezoelectric device, the interaction between the droplet and the device causes the deformation of the device, and thus the electric energy is generated. The amplitude and period of the piezoelectric device caused by the droplet impacting determine the power output of the device. Therefore, the power output of the device can be optimized by regulating the droplet impacting dynamics.

**Fig. 4:**
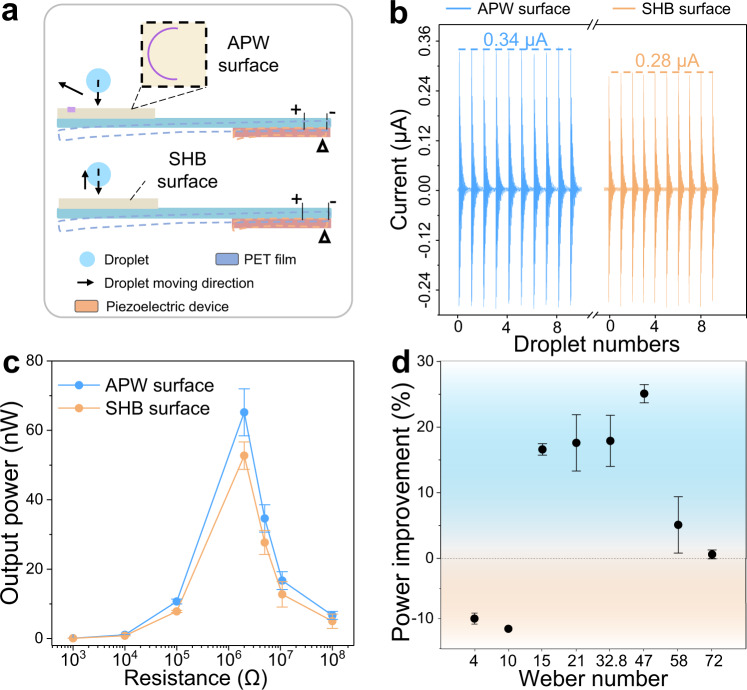
Enhancing the hydropower collection efficiency. **a** Schematic diagram of the piezoelectric devices equipped with APW (up) or SHB (down) surfaces. **b** Comparison of the current of ten droplets impacting on the piezoelectric devices equipped with APW or SHB surfaces. **c** Different output powers with different resistances. *We* = 47. **d** Power improvement ((*P*_-APW_−*P*_-SHB_)/*P*_-SHB_) under different Weber numbers. The external resistance is 2 MΩ. The error bars of the data in **c**, **d** are obtained from the s.d. of 7 independent experiments.

As discussed above, the Plateau–Rayleigh instability during droplet rebounding can be suppressed using patterned wettability substrates. The instability suppression is realized by regulating the liquid flow inside the droplet, which is governed by the asymmetric adhesion force from the substrate. According to Newton’s third law, the substrate is also subjected to an additional force from the droplet. Here we demonstrate that through rational utilization of the additional force, the hydropower collection efficiency using piezoelectric devices can be further improved, compared with that using a superhydrophobic surface that is considered as the most efficient surface^20,50^. Figure 4a is the scheme of the piezoelectric devices with APW and SHB surfaces. When the water droplet impacts on the beam, the impulse from the droplet causes the plate to deform and generate electricity. The short-circuit current of the device with APW surface is increased to 0.34 μA, compared to 0.28 μA from the piezoelectric device with the SHB surface (Fig. 4b). The increased current on the APW-based device results from the increased vibration frequency of the device, which is caused by the asymmetrical liquid flow inside the droplet (see Supplementary Figs. 12 and 13 and Supplementary Movie 2). The better performance in the output power is applicable for all the investigated load resistance range, as demonstrated in Fig. 4c and Supplementary Fig. 11. For a fixed external resistance of 100 KΩ, the maximum power output of the APW-based device shows an improvement by 36.5% than that of the SHB-based device (Supplementary Fig. 11). In addition, the influence of the Weber number on the power improvement is investigated, and the results are summarized in Fig. 4d. The APW-based device shows improvements for a wide range of Weber numbers (*We* ≥ 15). Detailed discussion of the dependence is provided in Supplementary Figs. 14 and 15 and Supplementary Discussion 3. This illustrates that by taking advantages of the asymmetrical droplet impacting dynamics, the hydroelectric power generation performance could be efficiently improved.

## Discussion

Compared with the homogeneous surface, the heterogeneous surface especially the patterned-wettability surface, is capable of precisely controlling the droplet retraction dynamics. Despite huge progress in the regulation of droplet dynamics on the patterned-wettability surface, precisely regulating the droplet Plateau–Rayleigh instability behavior still remains a challenge. Here, we realize the suppression of the Plateau–Rayleigh instability during droplet rebounding, through breaking the symmetry of droplet impact dynamics using patterned-wettability substrates. The instability-induced satellite drops generation is suppressed, and the droplet elongation is shortened. We reveal the mechanism that the wettability-pattern-induced asymmetric adhesion force regulates the liquid flow inside the droplet, and reduces the vertical velocity gradient of the rebounding droplet to suppress the instability. We propose that the liquid flow regulation strategy can be used to improve the droplet-based piezoelectric power generation efficiency, with the maximized improvement by 36.5% compared with the superhydrophobic piezoelectric device. It is anticipated that more counter-intuitive and useful functionalities could be developed by inducing asymmetry to the solid-liquid interactions.

## Methods

### Fabrication of substrates

The aluminum plates were cut into small flakes, and were corroded by Beck etchant (40 ml HCI (37 wt.%), 12.5 mL H_2_O and 2.5 mL HF (40 wt.%)) for 15 s. After blown drying with nitrogen and plasma treatment, the flakes were fluorinated by 1H, 1H, 2H, 2H-Perfluorodecyltriethoxysilane for 2 h at 80 °C. After fluorination, the flakes became superhydrophobic. Photomasks were used to prepare superhydrophilic patterns by UV radiation.

### Characterization

Scanning electron microscopy characterization was tested by SEM (F7500, JEOL). Dynamic contact angles were measured using the high-speed cameras: Phantom V12.1, Phantom VEO401L (Vision Research Inc.)

### Droplet impact experiments

The whole experimental set-up was placed at room temperature. Water drops (Diameter *D* = 2.1 mm) were generated from a fine needle equipped with a syringe pump from pre-determined heights. The diameter of different droplets is regulated by needles with different inner diameters. The dynamics of drop impact was recorded by high-speed cameras: Phantom V12.1, Phantom VEO401L (Vision Research Inc.).

Supplementary Method 1 details the calculation of the kinetic energy of the main droplets. Supplementary Discussion 1 details the droplet dynamics during the spreading and retraction process. Supplementary Note 1 details the kinetic energy of the main droplet *E*_k_ on diverse patterned-wettability surfaces. Supplementary Discussion 2 details the droplet impacting process under different *We* numbers and viscosities.

### Numerical methods and boundary conditions

The numerical calculations are conducted in software ANSYS Fluent. The dynamics of the water droplet impacting on the SHB and LPW surfaces are both simulated by using the coupled Level-set and Volume of Fluid (CLSVOF) method. The governing equations of the simulation method can be referred to the previous work^33,51^.

The water and air are simulated within a cylindrical domain with radius 6 mm and height 6 mm, which contains more than 4.6 million hexahedral cells generated by sweeping method after the grid independence test. To improve the accuracy of calculation, the near-wall grid is densified self-adaptively. The corresponding time step, 1.0 e–6 s, is of good efficiency and convergence. In the computational domain, the bottom surface is regarded as a non-slipping wall with different contact angles applied in different regions, and the other surfaces of the cylindrical domain are set as pressure outlet boundaries. The coupling equations of pressure and velocities were solved via the PISO (Pressure Implicit Split Operator) method. As for the two phases, the primary one is set as air, while the secondary one set as water. At the initial stage, the water droplet was modeled as a sphere with a downward impact velocity. All the parameters used in the simulations are list in the Supplementary Table 1.

Supplementary Method 2 details the parameters used in the simulations.

### Piezoelectric energy harvesting

Piezoelectric film (DT1-028K) was attached to the fixed end of a PET cantilever beam, and connected to the electrometer (Keithley 6514). The superhydrophobic and the patterned-wettability Aluminum flakes were pasted on the free end of the cantilever beam. The droplets impacted on the center of the Aluminum flakes to collect current and voltage signals.

Supplementary Discussion 3 details the droplet impacting process on the cantilever beams.

## Supplementary information

Supplementary Information

Description of Additional Supplementary Files

Supplementary Movie 1

Supplementary Movie 2

## Data Availability

The authors declare that the main data supporting the findings of this study are contained within the paper. Source data relating to Figs. 1d–f, 2b, d, 3b–d and 4b–d are provided as an additional file. All other relevant data are available from the corresponding author upon reasonable request. Source data are provided with this paper.

## Source data

Source Data

## References

[1] Xu WH (2020). A droplet-based electricity generator with high instantaneous power density. Nature.

[2] Lin S, Xu L, Chi Wang A, Wang ZL (2020). Quantifying electron-transfer in liquid-solid contact electrification and the formation of electric double-layer. Nat. Commun..

[3] Lin Z, Cheng G, Lee S, Pradel KC, Wang ZL (2014). Harvesting water drop energy by a sequential contact-electrification and electrostatic-induction process. Adv. Mater..

[4] Xu WH (2019). SLIPS-TENG: robust triboelectric nanogenerator with optical and charge transparency using a slippery interface. Natl. Sci. Rev..

[5] Liu YH, Andrew M, Li J, Yeomans JM, Wang ZK (2015). Symmetry breaking in drop bouncing on curved surfaces. Nat. Commun..

[6] Xiang C (2020). High efficiency and stability of ink-jet printed quantum dot light emitting diodes. Nat. Commun..

[7] Gu Z, Huang Z, Li C, Li M, Song Y (2018). A general printing approach for scalable growth of perovskite single-crystal films. Sci. Adv..

[8] Kim S, Moon M, Kim HY (2013). Drop impact on super-wettability-contrast annular patterns. J. Fluid Mech..

[9] Hao C (2015). Superhydrophobic-like tunable droplet bouncing on slippery liquid interfaces. Nat. Commun..

[10] Deng X, Mammen L, Butt HJ, Vollmer D (2012). Candle soot as a template for a transparent robust superamphiphobic coating. Science.

[11] Schutzius TM (2015). Spontaneous droplet trampolining on rigid superhydrophobic surfaces. Nature.

[12] Bird JC, Dhiman R, Kwon HM, Varanasi KK (2013). Reducing the contact time of a bouncing drop. Nature.

[13] Richard D, Clanet C, Quere D (2002). Surface phenomena: contact time of a bouncing drop. Nature.

[14] Liu J (2016). Guided self-propelled leaping of droplets on a micro-anisotropic superhydrophobic surface. Angew. Chem. Int. Ed..

[15] Song MR (2017). Controlling liquid splash on superhydrophobic surfaces by a vesicle surfactant. Sci. Adv..

[16] Liu Y, Tan P, Xu L (2015). Kelvin-Helmholtz instability in an ultrathin air film causes drop splashing on smooth surfaces. Proc. Natl Acad. Sci. USA.

[17] Yarin AL (2006). Drop impact dynamics: splashing, spreading, receding, bouncing. Annu. Rev. Fluid Mech..

[18] Liu J, Vu H, Yoon SS, Jepsen RA, Aguilar G (2010). Splashing phenomena during liquid droplet impact. Atomization Spray..

[19] Yokoi K (2011). Numerical studies of droplet splashing on a dry surface: triggering a splash with the dynamic contact angle. Soft Matter.

[20] Richard D, Quéré D (2000). Bouncing water drops. Europhys. Lett..

[21] Chen L, Xiao Z, Chan P, Lee Y, Li Z (2011). A comparative study of droplet impact dynamics on a dual-scaled superhydrophobic surface and lotus leaf. Appl. Surf. Sci..

[22] Fu T, Wu Y, Ma Y, Li HZ (2012). Droplet formation and breakup dynamics in microfluidic flow-focusing devices: From dripping to jetting. Chem. Eng. Sci..

[23] McGraw J, Li J, Tran D, Shi A, Dalnoki-Veress K (2010). Plateau-Rayleigh instability in a torus: formation and breakup of a polymer ring. Soft Matter.

[24] Wijshoff H (2018). Drop dynamics in the inkjet printing process. Curr. Opin. Colloid ..

[25] Damak M, Hyder MN, Varanasi KK (2016). Enhancing droplet deposition through in-situ precipitation. Nat. Commun..

[26] Guigon R, Chaillout JJ, Jager T, Despesse G (2008). Harvesting raindrop energy: experimental study. Smart Mater. Struct..

[27] Okumura K, Chevy F, Richard D, Quéré D, Clanet C (2003). Water spring: a model for bouncing drops. Europhys. Lett..

[28] Han X (2021). Slippery damper of an overlay for arresting and manipulating droplets on nonwetting surfaces. Nat. Commun..

[29] Liu Q (2021). The role of drop shape in impact and splash. Nat. Commun..

[30] Tang X (2021). Design of multi-scale textured surfaces for unconventional liquid harnessing. Mater. Today.

[31] Li H (2015). Splitting a droplet for femtoliter liquid patterns and single cell isolation. ACS Appl. Mater. Inter..

[32] Li H (2020). Droplet precise self-splitting on patterned adhesive surfaces for simultaneous multidetection. Angew. Chem. Int. Ed..

[33] Li H (2019). Spontaneous droplets gyrating via asymmetric self-splitting on heterogeneous surfaces. Nat. Commun..

[34] Liu X, Zhang X, Min J (2019). Spreading of droplets impacting different wettable surfaces at a Weber number close to zero. Chem. Eng. Sci..

[35] Li H (2021). Precise droplet manipulation based on surface heterogeneity. Acc. Chem. Res..

[36] Zhao Z (2019). Steerable droplet bouncing for precise materials transportation. Adv. Mater. Interfaces.

[37] Zhang T, Wu J, Lin X (2021). Lateral motion of a droplet impacting on a wettability-patterned surface: numerical and theoretical studies. Soft Matter.

[38] Ren H (2021). Lateral bouncing behavior of droplets on the wettability-patterned surface. CIESC J..

[39] Farshchian B, Pierce J, Beheshti MS, Park S, Kim N (2018). Droplet impinging behavior on surfaces with wettability contrasts. Microelectron. Eng..

[40] Mohammad Karim A, Fujii K, Kavehpour HP (2021). Contact line dynamics of gravity driven spreading of liquids. Fluid Dyn. Res..

[41] Wang Y (2019). Droplet sliding: the numerical observation of multiple contact angle hysteresis. Langmuir.

[42] Ilyas MA, Swingler J (2015). Piezoelectric energy harvesting from raindrop impacts. Energy.

[43] Wong V, Ho J, Chai A (2017). Performance of a piezoelectric energy harvester in actual rain. Energy.

[44] Jellard SCJ, Pu SH, Chen S, Yao K, White NM (2019). Water droplet impact energy harvesting with P(VDF-TrFE) piezoelectric cantilevers on stainless steel substrates. Smart Mater. Struct..

[45] Wong C, Dahari Z, Abd Manaf A, Miskam MA (2015). Harvesting raindrop energy with piezoelectrics: a review. J. Electron. Mater..

[46] Wang Z (2017). On Maxwell’s displacement current for energy and sensors: the origin of nanogenerators. Mater. Today.

[47] Guigon R, Chaillout JJ, Jager T, Despesse G (2008). Harvesting raindrop energy: theory. Smart Mater. Struct..

[48] Wang Z, Wang AC (2019). On the origin of contact-electrification. Mater. Today.

[49] Wang Y, Gao S, Xu W, Wang Z (2020). Nanogenerators with superwetting surfaces for harvesting water/liquid energy. Adv. Func. Mater..

[50] Vasileiou T, Gerber J, Prautzsch J, Schutzius TM, Poulikakos D (2016). Superhydrophobicity enhancement through substrate flexibility. Proc. Natl. Acad. Sci. USA.

[51] Sussman M, Puckett EG (2000). A coupled level set and volume-of-fluid method for computing 3D and axisymmetric incompressible two-phase flows. J. Comput. Phys..

