# Supplementary material for: Breaking the symmetry to suppress the Plateau–Rayleigh instability and optimize hydropower utilization
Source: Nat Commun. 2021 Nov 25;12:6899. doi: 10.1038/s41467-021-27237-0 (PMC8635411; doi:10.1038/s41467-021-27237-0)
Supplement: Supplementary file 1 — Supplementary Information [file 41467_2021_27237_MOESM1_ESM.pdf]

Supplementary Information for

Breaking the symmetry to suppress the Plateau-Rayleigh instability and optimize hydropower utilization

Zhipeng Zhao,[1,2] Huizeng Li,[1]* An Li,[1,2] Wei Fang,[3] Zheren Cai,[1,2] Mingzhu Li,[1,2] Xiqiao Feng[3]* and Yanlin Song[1,2]*

*Corresponding author. Email: lihz@iccas.ac.cn; fengxq@tsinghua.edu.cn; ylsong@iccas.ac.cn.

[1]Key Laboratory of Green Printing, Institute of Chemistry, Chinese Academy of Sciences (ICCAS)/Beijing Engineering Research Center of Nanomaterials for Green Printing Technology, Beijing National Laboratory for Molecular Sciences (BNLMS), Beijing, 100190, P. R. China.

[2]University of Chinese Academy of Sciences, Beijing, 100049, P. R. China.

[3]AML, CNMM and Department of Engineering Mechanics, and State Key Laboratory of Tribology, Tsinghua University, Beijing, 100084, P. R. China.

This PDF file includes:

Supplementary Methods 1 to 2
Supplementary Note 1
Supplementary Discussions 1 to 3
Supplementary Figs. 1 to 15
Captions of Supplementary Movies 1 to 2
References

Other Supplementary Materials for this manuscript include the following:

Supplementary Movies 1 to 2

Supplementary Method 1. Calculation of the kinetic energy of the main droplets.

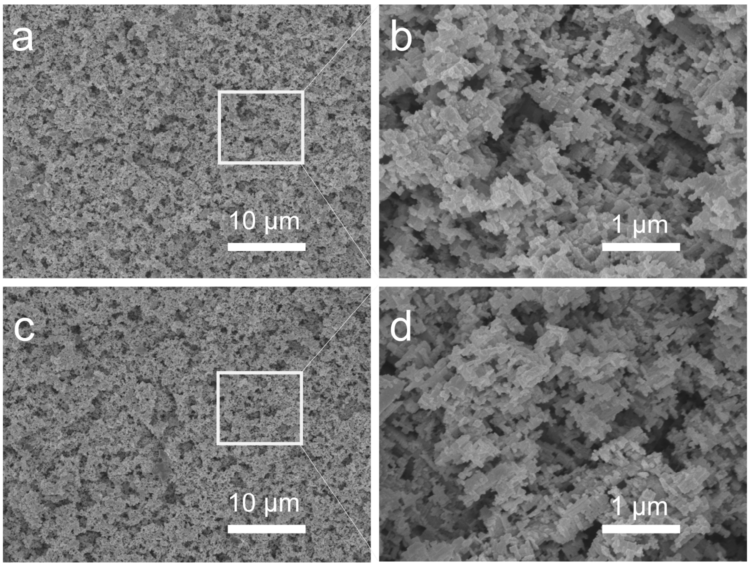

Supplementary Fig. 1. SEM characterization of SHB (superhydrophobic) region and SHP (superhydrophilic) region. (**a, b**) SHB region, (**c, d**) SHP region. The SHB and the SHP regions have similar micro-nanoscale structures.

As shown in Supplementary Fig. 1, both the SHB and the SHP surfaces have micro-nano structures of aluminum sheets, which correspond to the superhydrophobic and superhydrophilic property of the aluminum surface. The surface structure of the superhydrophobic region is similar to that of the superhydrophilic region.

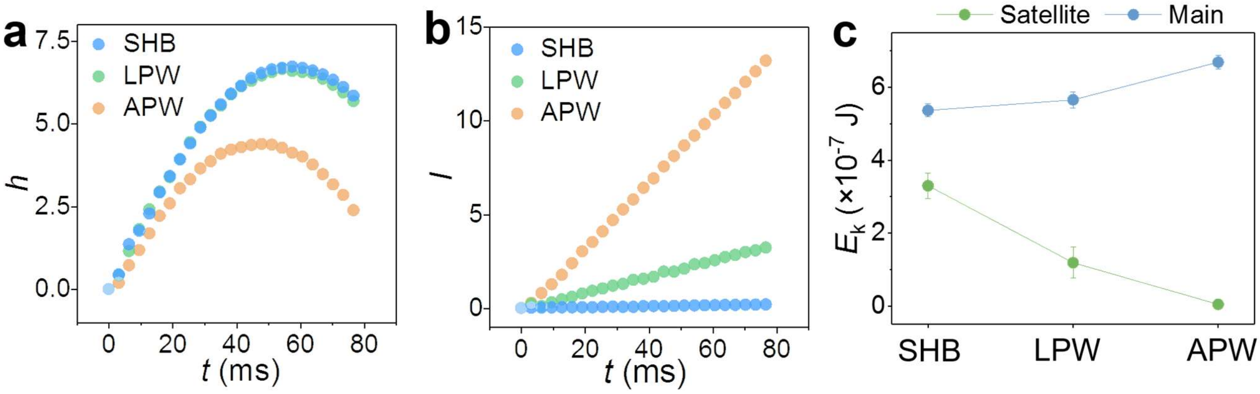

Supplementary Fig. 2. Variation of *h* (defined as the ratio of rebounding height *H* and the droplet diameter) and *l* (defined as the ratio of lateral displacements *L* and the droplet diameter) of the droplets from three surfaces. (**a**) *h*, (**b**) *l*, (**c**) the kinetic energy of the main droplet and the satellite drops. The error bars of the data in (**c**) are obtained from the s.d. of 5 independent experiments.

The evolution of the droplet rebounding height and the lateral displacement is shown in Supplementary Fig. 2a and 2b. In the vertical direction, the vertex of the droplet rebounding curve from the LPW surface is similar to that from the SHB surface, and is higher than that from the APW surface. Meanwhile, all these curves can be well-fitted by the freely upcast motion that is governed only by gravity (Supplementary Fig. 3). Meanwhile, when the main droplets reach the vertex of the rebounding curves, the surface area (surface energy) of the droplets from

the three surfaces are almost the same (see Fig. 1a-1c), indicating that the excess surface energy of the elongated droplets is dissipated and will not be converted into the kinetic energy of the droplet during the rebounding process.

In the lateral direction, the droplets only deviate on the APW and the LPW surfaces, with deviating more prominent on the APW surface (Supplementary Fig. 2b). The kinetic energy of the satellite drops generated from the Plateau-Rayleigh instability is shown in Supplementary Fig. 2c. The kinetic energy of satellite drops from the SHB surface is the largest, followed by that from the LPW and the APW surface.

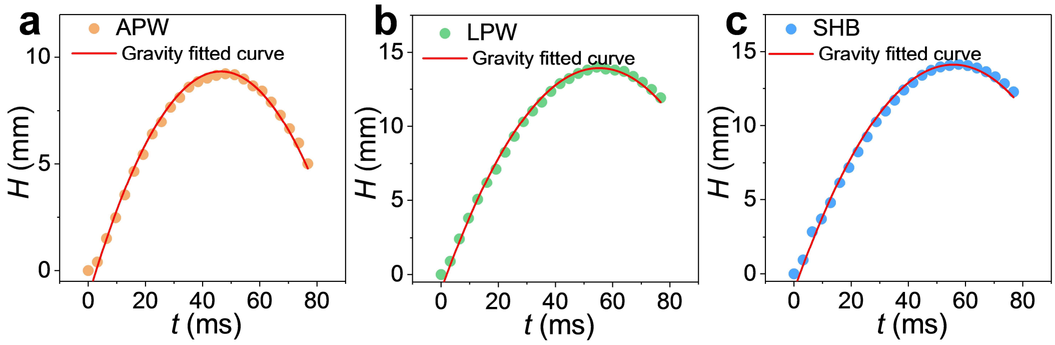

Supplementary Fig. 3. Comparison of the droplet trajectories in the vertical direction with the free upcast motion that is governed only by gravity. (a-c) Droplet trajectories of the APW, the LPW, and the SHB surface, respectively.

Calculation of E_k

First, we measure the velocity of the droplet at the moment of departure from the surface. Using a high-speed camera, the morphology of the droplet is precisely captured with a time-interval of 0.1 ms for adjacent frames. In the vertical (y axis) direction, the position of the droplet is obtained by averaging the positions of the top and bottom ends of the droplet. The trajectory of the droplet in the vertical direction is shown in Supplementary Fig. 3. The trajectory can be well fitted by the upcast motion curve that governed only by gravity, indicating that measuring the position of the droplet using this approach is reasonable. Accordingly, the velocity of the droplet in the vertical direction can be determined through the gravity fitting curve. Similar approach is used to calculate the droplet velocity in the lateral direction. Hence, we can get the total velocity of the droplet at the moment of departure from the surface.

Next, we measure the mass of the droplet. The shape of the droplet is elongated and irregular when departing from the surface. According to the conservation of mass, we measured the volume of the droplet at the peak of its upward motion, considering that it is spherical. The mass of the droplet can be obtained by multiplying volume with density.

The rebounding kinetic energy of the main droplet can be obtained by $E_k = \frac{1}{2}mv^2$.

Supplementary Method 2. Numerical methods and boundary conditions.

All the parameters used in the simulations are list in the **Supplementary Table 1**.

Supplementary Table 1. List of simulation parameters.

**Parameters**	**Values**	**Units**
Water drop diameter	2.1	mm
Drop impact velocity	1.5	$m\ s^{-1}$
Density of the water, air	998.0, 1.225	$kg\ m^{-3}$
Viscosity of the water, air	1.003e−3, 1.789e−5	Pa s
Gravitational acceleration	9.8	$m\ s^{-2}$
Surface tension of the water	72	$mN\ m^{-1}$
Width of the SHP pattern	200	μm
Contact angle of the SHB, SHP surface	160, 10	°

Remarks: SHP pattern and SHP surface stand for superhydrophilic pattern and superhydrophilic surface, respectively.

Supplementary Note 1. Droplets impact on different patterned-wettability surfaces.

During the analysis of the droplet receding dynamics, we propose the maximum net liquid flow rate u_{max} to evaluate the asymmetry of the liquid receding. Increasing the u_{max} can suppress the liquid instability and enlarge the main droplet rebounding kinetic energy. However, this principle should consider the adhesion force between the droplet and the superhydrophilic pattern, and is applicable to the patterned wettability substrates. Therefore, although the superhydrophobic surface has the lowest value of u_{max}, it has a relatively large value of E_{k}. For clarification, we designed a patterned wettability substrate consisting of a superhydrophilic line in the center of the pattern, as shown in Fig. 3b and 3c. When a water droplet impacts on the surface, it also has the lowest value of u_{max}, as well as a very small rebounding kinetic energy E_{k} (Fig. 3d).

Supplementary Discussion 1. The droplet spreading and retraction processes.

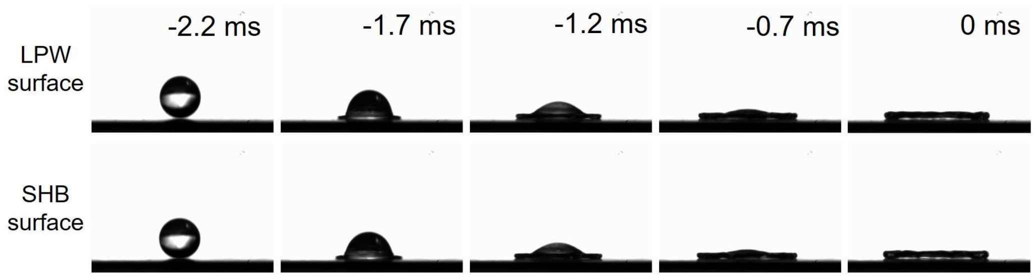

Supplementary Fig. 4. Snapshots of the droplet spreading process on the LPW and the SHB surfaces.

The droplets show indistinguishable spreading behaviors on the LPW and SHB surfaces, so we mainly focus on the droplet retraction stage.

In the droplet retraction stage, the asymmetrical lateral adhesion force from the patterned wettability substrate affects the internal liquid flow of the droplet, and makes the droplet retract asymmetrically. The greater the difference in the dynamic contact angles at the right and left sides of the droplet ($\theta_{left} - \theta_{right}$), the larger the lateral force (F) to droplets. The evolutions of the dynamic contact angles versus time on SHB and LPW surfaces are shown in Supplementary Fig. 5, the blue dots.

On the other hand, the contact line velocity represents the difficulty of retraction on the left and right sides of the droplet. When the contact line velocity on one side of the droplet reaches and remains zero, it means that this side is stuck by the hydrophilic pattern. At this time, the patterned-wettability substrate begins to generate asymmetrical lateral force to the droplet. Therefore, the longer the duration of the contact line velocity remains at 0, the longer the duration of the force generated by the substrate on the droplet. The evolutions of the contact line velocity versus time on SHB and LPW surfaces are shown in Supplementary Fig. 5, the orange dots.

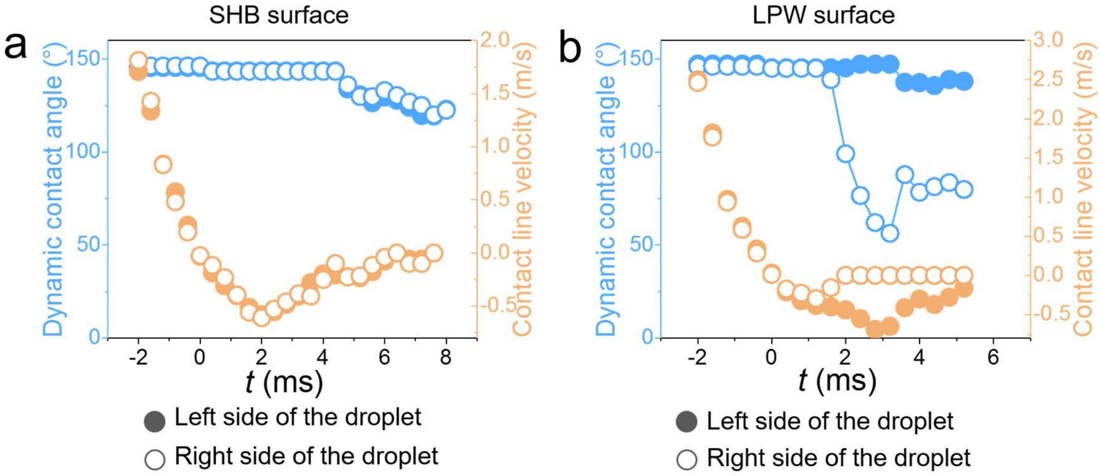

Supplementary Fig. 5. Dynamic contact angle and contact line velocity of the two surfaces. (**a**) SHB surface, (**b**) LPW surface.

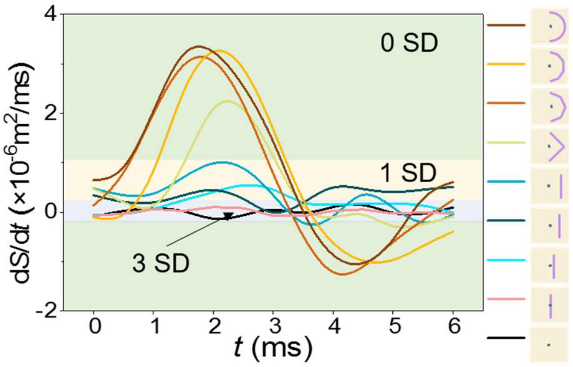

Supplementary Fig. 6. The variation of $\mathrm{d}S/\mathrm{d}t$ with time on different surfaces.

Supplementary Fig. 6 is roughly divided into 3 areas. $\mathrm{d}S/\mathrm{d}t$ of the SHB surface is close to 0 in the whole retraction process, as denoted by the blue area with 3 satellite drops (SD). The other two areas are the yellow area with 1 SD and the green area with 0 SD.

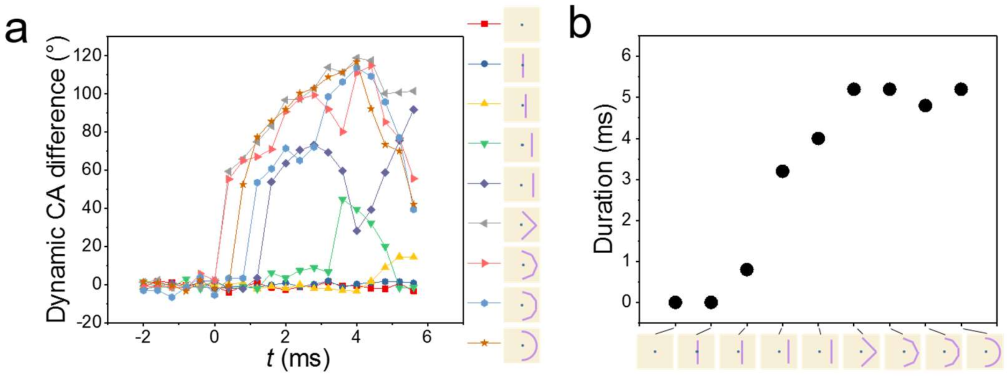

Supplementary Fig. 7. Dynamic contact angle (CA) difference of different pattern shapes. (**a**) Dynamic contact angle difference of different pattern shapes. (**b**) The duration for the dynamic contact angle difference of different pattern shapes ($(\theta_{left} - \theta_{right}) > 5°$).

After impacting on patterned-wettability substrates with different pattern shapes, the dynamic contact angles on the two sides of the droplet θ_{left} and θ_{right} vary greatly. For the LPW surface with the pattern center overlapped with the droplet impacting center, there is almost no difference of the dynamic contact angles on the two sides of the droplet, which means that the droplet impacting dynamics is symmetrical in the *x* direction. When the patterns are deviated from the droplet impacting center or using APW surfaces, θ_{right} and θ_{left} differ in the retraction stage. Both the dynamic contact angle difference ($\theta_{left} - \theta_{right}$) and the duration for the difference (Supplementary Fig. 7a and 7b) vary with the wettability pattern. The large difference and the long duration indicate that the wettability pattern exerts a large lateral force to the droplet, which enhances the asymmetrical internal liquid flow and suppress the Plateau-Rayleigh instability. For example, using a semicircle pattern, the droplet shows almost the largest contact angle difference and the longest duration of the difference, which correspond to the complete suppression of the droplet instability.

Supplementary Discussion 2. Droplets impacting under different *We* numbers and viscosities.

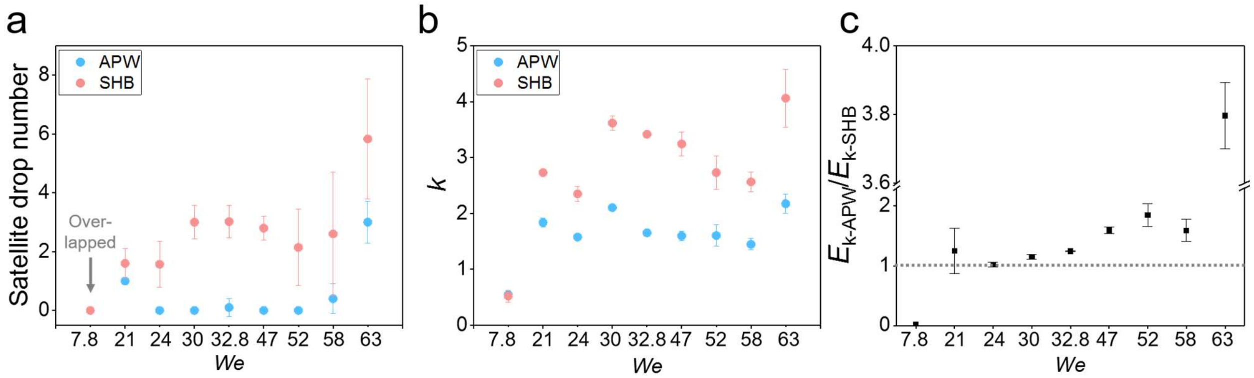

Supplementary Fig. 8. Droplets impacting under different *We* numbers. (**a**) Satellite drop number, (**b**) k (defined as the ratio of the main droplet elongation length and droplet diameter D), (**c**) $E_{k\text{-APW}}/E_{k\text{-SHB}}$ (the ratio of the kinetic energy of the main droplet rebound on the APW surface to the SHB surface) at different *We* numbers. The error bars of the data are obtained from the s.d. of 5 independent experiments

The generality of suppressing Plateau-Rayleigh instability by breaking the symmetry of surface wettability is then elucidated at different droplet *We* numbers. The results clearly illustrate that, for the droplets with *We* ranging from 21 to 63, the APW surfaces show a prominent effect on suppressing the instability of the rebounding droplet, with less satellite drops and shorter droplet elongation, compared with those from the SHB surface.

For small *We* such as *We* = 7.8, the droplet rebounds without satellite droplets on SHB surface, which is consist with the previous reports [1]. While the droplet is captured by the superhydrophilic patterns on the APW surface, due to the large solid-liquid adhesion force. For large *We* (e.g., *We* = 63), the droplets splash after impacting on both the SHB and the APW surfaces. However, in that case, the APW surface can still reduce the number of the satellite drops, compared with the SHB surface. The droplet instability suppression using APW surfaces can also be demonstrated by the enhanced rebounding kinetic energy of the main drops (Supplementary Fig. 8c).

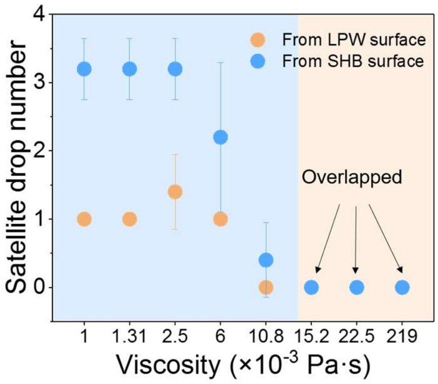

Supplementary Fig. 9. Satellite drops numbers of the droplets rebounding from two surfaces at different viscosities. The error bars of the data are obtained from the s.d. of 5 independent experiments.

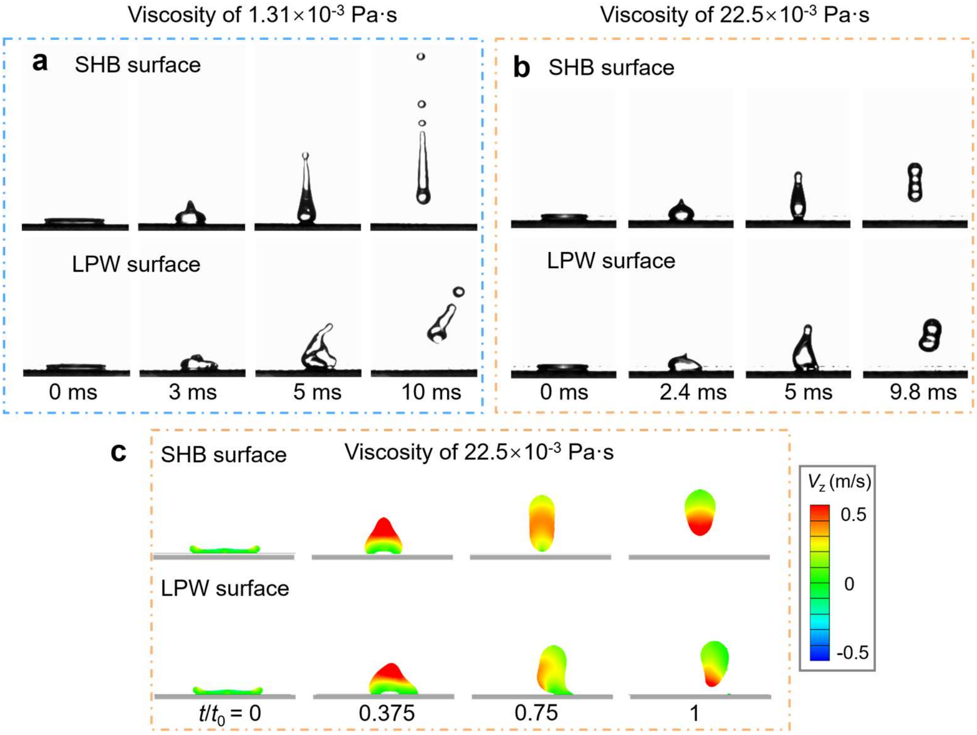

Supplementary Fig. 10. (**a**) Screenshots of droplets rebound with viscosity of 1.31×10^{-3} Pa·s. (**b**) Screenshots of droplets rebound with viscosity of 22.5×10^{-3} Pa·s. Different droplet viscosities are obtained by different ratios of glycerol and water. (c) Simulated results to show the evolution of the vertical (*z*-direction) velocity distribution in the cross-section of the droplets rebound with viscosity of 22.5×10^{-3} Pa·s.

To investigate the viscosity of the impacting droplet on the instability of the droplet impact dynamics during retraction and rebounding, we use aqueous solution of glycerol to adjust the droplet viscosity. The viscosity, surface tension, and density of the solutions with different ratios of glycerol and water are provided in Supplementary Table 2. The droplet impacting tests using these solutions are performed, and the results are summarized in Supplementary Fig. 9. At a small viscosity ($\leqslant 10.8\times10^{-3}$ Pa·s), the retraction and rebound of the droplet on both the LPW and the SHB surfaces are similar to those of deionized water, as shown in Fig. 1 and Supplementary Fig. 10a. At a large viscosity ($\geqslant 15.2\times10^{-3}$ Pa·s), there is great viscous dissipation in the droplet impacting and retraction process. At the same time, we selected a viscosity of 22.5×10^{-3} Pa·s for numerical

simulation. As shown in Supplementary Fig. 10c, the numerical simulation results are in good agreement with the experimental results in Supplementary Fig. 10b. The simulation results show that under high viscosity, the vertical velocity gradient of the droplets is small, which is not enough to produce satellite droplets. This indicates that during the internal flow of the droplet, due to the increase in viscosity, the viscosity loss greatly increases, which consumes excess kinetic energy of the droplet [2,3], thereby inhibiting the generation of satellite droplets. Therefore, there will be no satellite drops both on SHB and LPW surfaces.

Supplementary Table 2. Parameters of glycerol aqueous solution with different glycerol mass fractions.

Weight fraction of glycerol	Viscosity (×10^{-3} Pa·s)	Surface tension (mN/m)	Density (g/cm^3)
0	1	72.8	1
10%	1.31	72.1	1.03
30%	2.5	71	1.07
50%	6	69.5	1.13
60%	10.8	68	1.16
65%	15.2	67.5	1.17
70%	22.5	67.1	1.18
90%	219	66.3	1.24

Supplementary Discussion 3. Droplets impacting on the cantilever beams.

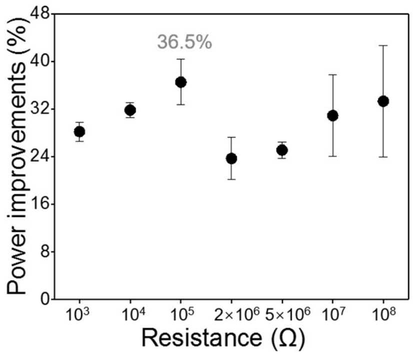

Supplementary Fig. 11. Power improvements under different resistances. The maximum power improvement (($P_{\text{-APW}}$-$P_{\text{-SHB}}$)/$P_{\text{-SHB}}$) is 36.5%, where the external resistance is 100 KΩ. The error bar of the output power is obtained from the s.d. of at least 7 independent experiments.

The cantilever beam used in our work has the eigen vibration frequency f_{b} of 104.3 Hz. f_{b} is calculated as $f_{\text{b}} = \frac{3.52}{2\pi}\sqrt{\frac{EI}{mL^3(1-n^2)}}^{4}$, where m, L, I, E, and n are the beam mass, length, area moment of inertia ($I = bc^3/12$, where b is the width and c is the thickness of the beam.), Young's modulus ($E_{\text{-PET}}$ = 3700 MPa), and the Poisson's ratio ($n_{\text{-PET}}$ = 0.3), respectively.

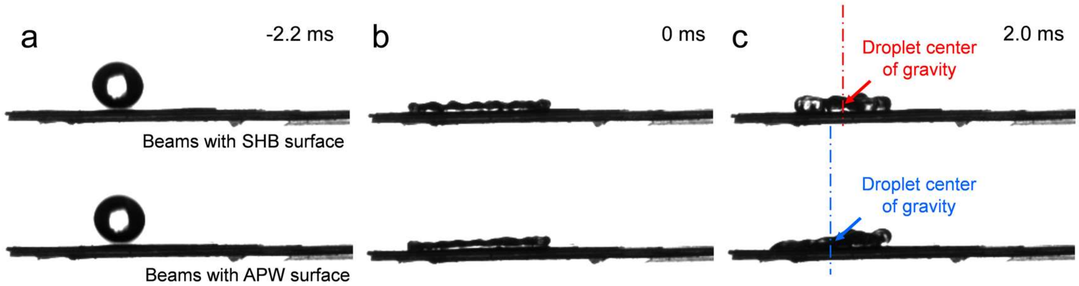

Supplementary Fig. 12 Droplet impact physics of the droplets and the cantilever beams. (a) Droplets contacting with the beams. (b) Maximum spreading of droplets. (c) Comparison of force arm in the process of droplet retraction.

After impacting on the cantilevers, the droplets exhibit similar spreading behaviors on SHB and APW surfaces (Supplementary Fig. 12a, 12b, and Supplementary Movie 2), meaning that the wettability pattern on the APW surface shows negligible influence to the droplet spreading. At the droplet retraction stage, the droplet recedes symmetrically on the SHB surface, while the center of gravity remains fixed in the whole retraction stage. However, on the APW surface, the droplet recedes asymmetrically due to the lateral adhesion force from the superhydrophilic pattern, making the gravity center the droplet shifting to the left (Supplementary Fig. 12c and Supplementary Movie 2). As a result, the force arm of the cantilever beam is extended, and the torque applied to the cantilever beam is increased.

The centers of gravity of the droplets are obtained by averaging the positions of the left and right ends of the droplets (Here we focus on the horizontal center of the droplet), which is similar to the center of gravity measurement method in Supplementary Method 1.

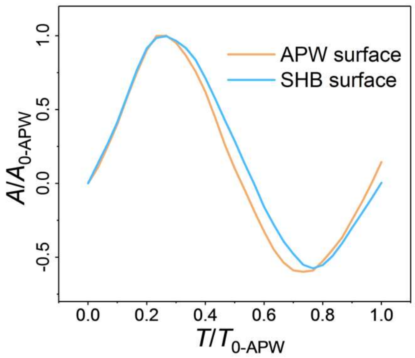

Supplementary Fig. 13. The amplitude of two kinds of cantilever beam in one period after one droplet impact, respectively.

For a piezoelectric device, the generated voltage is decided by the amplitude of the deformation [5,6]. In a capacitive element, the current is proportional to the change rate of the voltage. Increasing the vibration frequency of the device (or shortening the vibration period) will enlarge the change rate of the voltage, which enlarges the current. Therefore, compared with the SHB surface, the vibration frequency of the cantilever beam is obviously promoted, as shown in Supplementary Fig. 13, which corresponds to the larger current peaks on APW surface (Fig. 4b). Consequently, the output power of the droplet is increased compared with SHB surface.

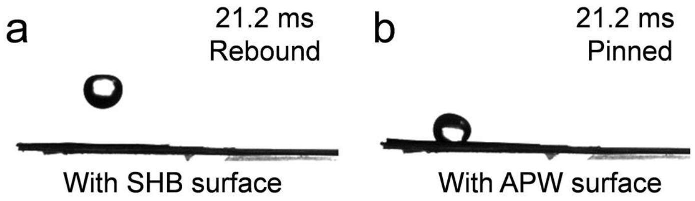

Supplementary Fig. 14. Rebounding of droplets after impacting on the beams at *We* = 4. (**a**) Beams equipped with SHB surface. (**b**) Beams equipped with APW surface.

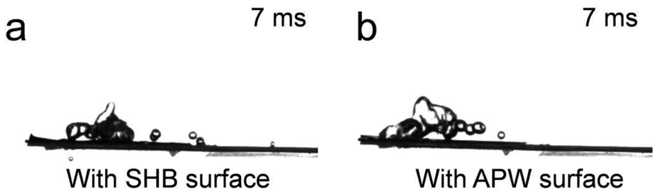

Supplementary Fig. 15. Rebounding of droplets after impacting on the beams at *We* = 72. (**a**) Beams equipped with SHB surface. (**b**) Beams equipped with APW surface.

The effect of the Weber number ($We = \rho V^2 R/\gamma$, where ρ is the liquid density, V is the liquid density, R is the droplet radius, and γ is the liquid surface tension) on the power output of the device, is investigated to provide more application scenarios for applications such as harvesting renewable sources like raindrops. As summarized in Fig. 4d, for a large Weber number range ($We \geqslant 15$), the power output of the beams equipped with an APW surface are larger than that with a SHB surface.

For small *We* numbers, i.e. $We = 4$, the droplet will be pinned by the superhydrophilic pattern on the APW surface due to the small kinetic energy (Supplementary Fig. 14). In that case, the power output is smaller than

that using a superhydrophobic surface. For large *We* numbers, i.e. *We* = 72, the droplets splash after impacting on both the superhydrophobic and the APW surfaces, as shown in Supplementary Fig. 15. The droplet splashing behaviors are almost the same for these surfaces, therefore generating similar power output.

Supplementary References

1 D. Richard&D. Quéré, Bouncing water drops. *Europhys. Lett.* **50**, 769 (2000).

2 D. Bartolo *et al.*, Bouncing or sticky droplets: Impalement transitions on superhydrophobic micropatterned surfaces. *Europhys. Lett.* **74**, 299-305 (2006).

3 K. Okumura, F. Chevy, D. Richard, D. Quéré&C. Clanet, Water spring: A model for bouncing drops. *Europhys. Lett.* **62**, 237 (2003).

4 P.B. Weisensee, J. Tian, N. Miljkovic&W.P. King, Water droplet impact on elastic superhydrophobic surfaces. *Sci. Rep.* **6**, 30328 (2016).

5 S.C.J. Jellard, S.H. Pu, S. Chen, K. Yao&N.M. White, Water droplet impact energy harvesting with P(VDF-TrFE) piezoelectric cantilevers on stainless steel substrates. *Smart Mater. Struct.* **28**, 095002 (2019).

6 R. Guigon, J. Chaillout, T. Jager&G. Despesse, Harvesting raindrop energy: experimental study. *Smart Mater. Struct.* **17**, 015039 (2008).

Captions for Supplementary Movies 1-2

Supplementary Movie 1: Symmetrical and asymmetrical droplet impact dynamics.

Supplementary Movie 2: Droplets impacting on piezoelectric devices at $We = 47$.
