# Supplementary material for: Breaking the symmetry to suppress the Plateau–Rayleigh instability and optimize hydropower utilization
Source: Nat Commun. 2021 Nov 25;12:6899. doi: 10.1038/s41467-021-27237-0 (PMC8635411; doi:10.1038/s41467-021-27237-0)
Supplement: Supplementary file 2 — Description of Additional Supplementary Files [file 41467_2021_27237_MOESM2_ESM.pdf]

**Description of Additional Supplementary Files**

**File Name:** Supplementary Movie 1
**Description:** Symmetrical and asymmetrical droplet impact dynamics.

**File Name:** Supplementary Movie 2
**Description:** Droplets impacting on piezoelectric devices at We = 47.
